# Experience in Puerperal Eclampsia

**Published:** 1877-08

**Authors:** A. J. Jessup

**Affiliations:** Westtown, New York


Experience in Puerperal Eclampsia.—*By A. J. Jessup, M.D., of Westtown, New York.*—In my experience of eclampsia in the puerperal state, I have observed the following facts:

1. There has always been albuminuria, or, more strictly speaking, uræmia; although undiscovered, from lack of thoroughness in examination.

2. In a majority, the convulsive seizures occur between the fifth and seventh month of utero-gestation.

3. That a firm fiber with an adipose temperament are the class, *a priori*, in which we would most frequently expect convulsions.

4. When setting in after labor, with complete or nearly complete suppression of urine, death is inevitable.

5. As a nearly exceptionless rule, when labor has declared itself, empty the uterus as soon as practicable, no matter at what period of pregnancy occurring.

6. When there is no declared action of the womb refrain from interference, unless at full term, when, if convulsions persist, empty the uterus.

7. Almost always when occurring prior to full term the life of the fœtus is destroyed; occasionally this happens at full term. These results being due to convulsions or blood-poisoning to the fœtus, or both.

8. When conditions are favorable, extract blood largely.

9. Chloroform must always be an adjuvant to venesection, or when the former is not practicable, give to control the fits; the method will be mentioned further on, when mentioning cases.

10. The use of some free evacuant to bowels, skin and kidneys, is most imperatively demanded, the object being, as far as possible, to unload the blood of the poisonous principles, the elements of the urine.

My object in this report is merely to give the aspects and mode of management of my cases, and not to enter into a pathological history of the disease; to offer them to your readers as my quotum toward the aggregate of experience in this interesting affection.

*Case* 1.—Mrs. J., reported by Dr. J. H. Thompson, May 28, 1870, in the *Medical and Surgical Reporter* of that date. Patient was my wife; the main peculiarity in treatment was the extraction of blood from the temporal artery, owing to the excessive œdema of the arm concealing the veins, and rendering venesection impossible, together with a concurrent dislocation of the humerus, caused by a fall from her couch during the first convulsion.

*Case* 2.—Patient was the same lady, aged twenty-eight, at full term, July 13, 1871. Fits occurred at full term, after delivery. Albumen first seen at sixth month; urine was tested daily up to day of confinement, showing increasing proportions of albumen, until it became nearly solid in the test tube; kept up a thorough dietetic, diuretic, and moderately laxative treatment, which, however, did no more than prevent anasacra, and kept up a moderate flow of the urine, without relieving the renal oppression. A consultation was proposed, with the view of bringing on premature delivery, but was objected to by patient; she preferred to assume all risks in the hope of having a living child. Here we cannot too much admire the heroic unselfishness of mothers, who prefer to hazard life itself rather than forego the prospect of maternity and all its hallowed affection, which extends even to the child yet unborn. After a tedious labor of nine hours, tedious from rigidity of the os, was delivered of a still-born child at full term, weighing eight pounds. One-half hour afterward seized with clonic convulsions, affecting chiefly head, fece and arms. Pulse feeble, 140; weak; skin cold; countenance purple; there did not seem power in the feeble heart to propel the blood to the surface, therefore venesection was without results. Two medical friends saw her, agreeing that her condition was one of uræmic oppression of the nerve-centers. The slight convulsions were warded off by chloroform, and, indeed, they formed no material element in her danger; she remained semi-comatose during the night, arousing at intervals, taking cold water freely; no urine; bowels would not respond to jalap, elaterium, or two drops of croton oil. On using the catheter obtained about one-half ounce of urine, very dark, of the consistence of molasses; no more secreted while she lived. There seemed no response from the nerve-centers of organic life, to any stimuli employed; pulse, seven hours after first convulsion, was beyond computation; coma complete. The final scene ended fifteen hours after the first fit, and twenty-seven hours after commencement of labor. Toward the end congestion of the lungs was present very markedly.

This mode of death from uræmia is mentioned by Alonzo Clark. Here was a case in which the nerve-centers, brain, medulla, and all the ganglia of organic life were overwhelmed by the blood-poison everywhere present throughout the tissues, supplied by the vitiated fluid, whose deadly effects were observed even on the fœtus in utero, failing, as I believe, to support the life there, on account of its poisonous ingredients. Being insufficient to supply the medulla and par-vagi, they, in their turn, failed to stimulate the heart and lungs, as evidenced by the damming of blood on the right side of the heart, and slowness of the respiratory actions. By the breathing during the last twelve hours of life, it would seem that the par-vagi were insufficient, by its inhibitory function, to keep up normal respiration, the character of the breathing being thus: becoming slower, and at longer intervals until scarcely perceptible, then there would come a deep gasp, then the process repeated as before; as if nature required to be supplemented by the voluntary act in order to re-establish her function, which, when removed, respirations came faint, and fainter, until the vital function seemed almost submerged by the letheal tide, before a voluntary effort would come to temporarily restore the function, until final congestion and insensibility marked impending dissolution.

Another effect of this blood poison is shown in the failure of the sympathetic to respond to cathartic action, the kidneys to diuretic, resulting in engorgement, congestion, inflammation, and final suppression of secretion.

The skin acted profusely, but only by a watery exudation, without smell or color, and valueless as an outlet of poisonous materials.

I have dwelt thus long upon this case, because of the deep personal interest, and also hoping to throw some light upon those physiological and pathological actions of which I have seen no adequate explanations in the writings or teachings on the subject of uræmia, and hoping they may aid in throwing some light on the phenomena of other cases of this disease. I pass on to Case 3, merely hinting that those gentlemen who claim never to have lost a case of eclampsia, who are drawn up in battle array against those who have, should consider that it is their good luck, and hoping that, should the time come when they will meet one of these helpless cases, they may feel more charitable to their more unfortunate brethren.

Puerperal eclampsia; primipara, seven months, æt twenty-two, with delivery by craniotomy. E. T.; called December

31, 1874; of robust physique, in seventh month; being absent, Dr. Whitaker was called, both being present at 3 P.M.; had had four convulsions a few days previously; complained of headache, vertigo and general malaise; with the Doctor's help withdrew twenty-eight ounces from the arm; at 4.30 P.M. a fifth fit occurred; kept her under chloroform until one hour had passed; the sixth fit occurred. The Doctor left; I kept her under anæsthetic nearly continuously for three hours, giving thirty grains potassium bromide every thirty minutes; and half grain morphia, subcutaneously; not repeated; no more convulsions; urine highly albuminous; prescribed inf. dig., with an active purge.

January 1. Pulse 90, temperature 98: had rested well; bowels had acted freely; urine normal in quantity, albuminous.

From this date until January 6th was about the house; on the evening of this date was taken in labor, with some pains until 8th, when they began more actively; on being called, was again absent; Dr. Whitaker saw her; found os would admit two fingers; gave a full dose fl. ext. ergot; left. When I saw her the os was as large as a half-dollar; uterus seemed in a state of tonic contraction, with rigid os. Feeling certain that the ergot was acting badly, and having some anxiety that my patient should do well, having passed through one siege of eclampsia, I regretted the dose having been given; however, it would do no good to lament, so I tried every known method to relax the rigid os without avail. At 2 A.M. the pains continuing severe, without further dilatation, patient becoming exhausted, with a wild look in her eyes, what I most dreaded happened, one of the most severe, long-continued convulsions I ever witnessed; bled to sixteen ounces; chloroform; a second fit occurred in thirty minutes. I kept her fully under chloroform; sent for Dr. Whitaker and Dr. Haynes, who arriving, agreed with me that, as it was unsafe to attempt delivery with forceps, we should proceed to deliver by craniotomy at the earliest possible moment, as we had great reasons to fear a third convulsion like the last. Dr. Whitaker gave the anæsthetic; Dr. Haynes aiding by supporting abdomen, I proceeded to perforate; guarding the lips of the os by the fingers of the left hand, I succeeded in breaking down the cranium and delivering. Uterus contracted well; patient, after some fever and debility, made a good recovery. There was no doubt about the life of the child being extinct; motion had not been felt for more than twenty-four hours past.

I do not remember having heard of a case where craniotomy was required for such a cause. This case required immediate action; delivery must be had without delay, all the medical men present agreeing that the brain could not withstand the pressure of another convulsive seizure.

*Case* 4.—Puerperal uræmia, with impending eclampsia. A. Mc——, aged twenty-seven; primipara; adipose; plethoric; had always had perfect health; had been suffering with headache, with abdominal pains, referable to region of the stomach, and nausea, for one week previous to my visit. I was called on account of a violent and persistent headache. She described pain "as if caused by a nail being driven over right eye." I tested urine, and found it highly albuminous. While engaged in this, patient called me to the bedside. She sat up, saying that she could see but half of my face, while the supraorbital pain became so intense as to cause her to cry out with agony. Without losing a moment, I took blood without stint, not caring for measurements. I bled until she fell over on the bed, fainting, in all a large wash-basin nearly full—about three pints. This relieved the intra-cranial pressure, as also the pain and defect of vision. The usual evacuants, with sedatives, were prescribed. At the end of three days I was summoned to attend her in miscarriage, death of the fœtus having taken place, and, of course, the uterus proceeded to expel the foreign substance. This must have been a result of uræmia. This case will also illustrate the necessity of extreme caution in dealing with pregnant primiparæ. We should examine the symptoms with extreme care, to the end that we be enabled to step between the patient and danger or death, and so happily ward off either.

*Apropos.* Since we have so powerful an agent for relief, when used in proper cases, it would be well to remind those physicians who, in following modern fashions too much, neglect this important therapeutic agent, that in seeking a substitute in chloral, veratrum, aconite, and other deadly agencies, they are handling two-edged swords, which oftener sever the

"silver cord" than relieve disease, and would call their attention to the report of a case from the pen of W. H. Parrish, in the *Reporter*, February 23, 1876, Case 3. The treatment there needs no comment.

*Case* 5.—Mrs. DeG., age twenty-three, primipara, full term, of delicate frame, but healthy; albuminuria; venesection, twelve ounces. In this case chloroform controlled fits easily (as indeed it did in all my cases subsequent to venesection). Labor set in three days after, with the birth of a healthy child.

I will conclude with a description of the method which I have found most useful in administering chloroform.

The fits generally recur at exactly equal intervals. For instance, if the first two are fifteen minutes apart, they will continue to recur at the end of each fifteen minutes; if half-hour apart, one hour, and so on, unless the order of succession is broken by your efforts to subdue, or some change takes place in the phase of the attack. The practical application of this observation will be appreciated when giving chloroform; it would be unwise to keep a patient under chloroform from hour to hour, so my plan is to watch the clock, and when the time approaches when we may certainly expect a convulsion, I bring the patient fully under, keep her so until the time is past, then discontinue until the next, and so on, when, after four or five hours of such treatment, your patient will be secured for a considerable time, say twenty-four or forty-eight hours, from a recurrence of the fits; generally the period of immunity persists until the onset of labor. There is one little caution needed. We will suppose you have controlled three fits; in doing so you have broken the order of their regularity. You then relax your vigilance, and are surprised by a fourth.

Now, to make sure that there shall be but one fit after I pursue the method as above, with the further watchfulness of commencing the anæsthetic at the slightest signs of restlessness, staring, etc., which signs are generally the premonitions of a convulsion. Adjuvants, as mentioned above, will carry your patient on until labor supervenes. If it has set in, the uterus must be emptied as soon as practicable.—*Medical and Surgical Reporter.*

